# Tomato yellow leaf curl Reunion virus: a novel tomato-infecting monopartite begomovirus from Reunion Island

**DOI:** 10.1007/s00705-025-06345-y

**Published:** 2025-07-05

**Authors:** Pierre Leclair, Murielle Hoareau, Evelyne Parvedy, Elise Quirin, Clarisse Clain, Estelle Roux, Janice Minatchy, Pierre-Yves Teycheney, Pierre Lefeuvre, Jean-Michel Lett

**Affiliations:** 1 CIRAD, UMR PVBMT, Pôle de Protection des Plantes, 97410 Saint-Pierre, La Réunion France; 2 Institut Polytechnique UniLaSalle Rouen, Mont-Saint-Aignan, France; 3 FDGDON, Clinique du Végétal®, 97410 Saint-Pierre, La Réunion France; 4 SCA Fruits de La Réunion, 7 chemin de l’Océan, 97450 Saint-Pierre, La Réunion France; 5 FDGDON, 97460 Saint-Pierre, La Réunion France; 6 College of Agriculture, CIRAD, UMR PVBMT, Can Tho University, Can Tho, Vietnam

## Abstract

This is the first description of the complete genome sequence of a new monopartite begomovirus isolated from tomato leaves with symptoms of tomato (yellow) leaf curl disease collected on Reunion Island, for which we propose the name "tomato yellow leaf curl Reunion virus" (TYLCREV). Phylogenetic analysis showed that TYLCREV belongs to the Old World monopartite begomoviruses and is most closely related to tomato yellow leaf curl virus (TYLCV). The genome of TYLCREV displays evidence of interspecies recombination and is composed of a portion (52%) that is closely related to TYLCV (>97.6% sequence identity) and a portion (48%) that is closely related to begomoviruses that have been found in tomato plants in Madagascar (> 91.6% sequence identity). The discovery of TYLCREV in diseased tomato plants in Reunion calls for epidemiological surveillance and further investigations to assess the prevalence and risks associated to this new virus in Reunion and beyond.

**Supplementary Information:**

The online version contains supplementary material available at 10.1007/s00705-025-06345-y.

Since the late 1980s, begomoviruses (genus *Begomovirus*, family *Geminiviridae*) have increasingly threatened the cultivation of important crops worldwide, including vegetable, staple, and cash crops [14]. Begomoviruses have a single-stranded DNA monopartite (DNA-A-like component) or bipartite (DNA-A and -B components) genome [6]. Recombination has been shown to play a key role in their emergence, evolution, speciation, and adaptation [6, 10].

Tomato (yellow) leaf curl disease (ToLCD-TYLCD) is predominantly associated with monopartite begomoviruses, including the worldwide emerging tomato yellow leaf curl virus (TYLCV, *Begomovirus coheni*). TYLCV originated in the Middle East and spread to the Mediterranean basin, and then to the rest of the world [9]. Other indigenous monopartite begomoviruses have been described in tomato (*Solanum lycopersicum*) in several sub-Saharan African countries, and on the islands of the South-West Indian Ocean (SWIO), especially Madagascar [4, 8, 17]. In Reunion, TYLCD has been associated with successive biological invasions of the Mild (TYLCV-Mld; [16] and Israel (TYLCV-IL; [5]) strains of TYLCV, which ended up coexisting in tomato fields [15].

In February and August 2024, leaf samples from two tomato plants (C24-558; C24-1719) showing severe curling symptoms with or without interveinal yellowing resembling those caused by ToLCD-TYLCD (Supplementary Fig. S1) were collected in two greenhouses located in the distinct areas of St. Louis and Le Tampon, respectively, in the south of Reunion (Supplementary Table S1). Total DNA was extracted using a DNeasy Plant Mini Kit (QIAGEN, France), following the manufacturer’s instructions. PCR assays were performed using a set of degenerate primers (FD382/RD1038) [11] designed to amplify the coat protein gene (ORF V1) of Old World begomovirus DNA-A-like components. PCR products of the expected sizes were obtained from both samples, suggesting the presence of an Old World begomovirus. Sequences of 569 bp were obtained for both amplicons by direct sequencing in both directions (Supplementary Table S1). Their sequences shared 99.6% identity with each other and were most closely related (BLASTn, 90.12% identity) to a sequence from the monopartite begomovirus tomato leaf curl Madagascar virus (ToLCMGV; AJ865339; species *Begomovirus solanummadagascarense*). No DNA-B or betasatellite molecules were detected by PCR using PBL1V2040-PCRC1 and Beta1/Beta2 primer pairs, respectively [2, 18] (Supplementary Table S1).

For the purpose of taxonomic classification, the full-length viral genome was amplified by rolling-circle amplification from the total DNA extracted from both leaf samples, using Phi29 DNA polymerase. Amplification products were digested with *Eco*RV endonuclease, and the putative monomeric full-length DNA molecules that were obtained (~3kb) were cloned into the pJET1.2 vector and sequenced by standard Sanger sequencing using a primer-walking approach.

Two complete DNA-A-like sequences 2,780 nucleotides in length were obtained from samples C24-558 (accession number PV126637) and C24-1719 (PV126638). The sequences exhibited features typical of Old World begomoviruses, with six open reading frames (ORFs) encoding proteins > 80 aa: two (V1 and V2) on the viral strand and four (C1, C2, C3, and C4) on the complementary strand. The intergenic regions contained a characteristic inverted repeat stem-loop structure including the conserved nonanucleotide sequence TAATATTAC and a putative Rep protein high-affinity binding site **GGGTC** [1]. The corresponding iteron-related domain (Rep N-terminal domain) was identified as MAPP**KRFLIN**. Alignment of the complete DNA-A-like sequences of the two isolates and selected Old World monopartite begomoviruses originating from Africa and the Mediterranean basin was performed using MUSCLE in MEGA X [7]. Maximum-likelihood (ML) phylogenetic trees were constructed using MEGA X [7] with automatic selection of the best-fit nucleotide evolution model. Pairwise identity comparison of these nucleotide sequences was performed using SDT v1.2 with pairwise deletion of gaps [13]. The DNA-A-like sequences PV126637 and PV126638 were very similar to each other (99.7%) and shared the highest nucleotide sequence identity (88.8 and 89.0%, respectively) with that of a TYLCV-Mld isolate from Reunion (AJ865337) (Fig. 1). In accordance with the begomovirus species demarcation criteria (91% nucleotide sequence identity in the DNA-A and DNA-A-like nucleotide sequences [2]), the two Reunionese isolates (named RE-558EC-24 and RE-1719EA-24) represent a new tomato-infecting monopartite begomovirus, for which we propose the virus name ‘‘tomato yellow leaf curl Reunion virus’’ (TYLCREV) and the species name "*Begomovirus solanumflavusreunionense*".

**Fig. 1:**
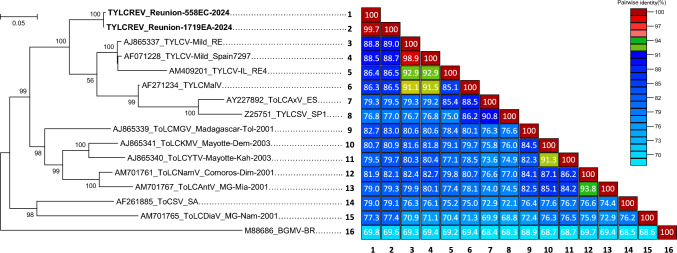
Maximum-likelihood phylogenetic tree of the complete DNA-A-like nucleotide sequences of tomato yellow leaf curl Reunion virus (TYLCREV) isolates RE-558EC-24 and RE-1719EA-24 and selected Old World monopartite begomoviruses originating from Africa, the Mediterranean basin, and the Middle East. Only bootstrap values higher than 70% are shown (1,000 replicates). Each coloured cell represents the percentage sequence identity between the two sequences. The correspondence between colours and pairwise identity is indicated by the colour scale on the right. According to the species demarcation threshold for begomoviruses, red, green, and blue colours indicate pairs of the same strain (≥94%), same species (≥91%), and different species (<91%), respectively. For begomovirus abbreviations, see Supplementary Table S2

Using RDP4 software [12] with default settings, we analyzed the sequence alignment including the complete genome sequences of TYLCREV and the potential parental monopartite begomovirus sequences to search for evidence of recombination in the TYLCREV genomes. We detected two recombinant fragments (a and b) totaling 48% of the TYLCREV genomes (Fig. 2A). Fragments a and b were found to be closely related to ToLCMGV (AJ865339, 91.6%) (Fig. 2C) and tomato leaf curl Diana virus (ToLCDiaV; AM701765, 97.5%) (Fig. 2D), respectively, two begomoviruses that have been found in tomato plants in Madagascar [8]. The non-recombinant part (genomic background) covered 52% of the TYLCREV genomes and was most closely related to TYLCV-Mld, with 98.4% identity (BLASTn, AJ865337 and AB116632; Fig. 2B). The ML tree confirmed that the complete nucleotide sequences of the two TYLCREV isolates form a distinct branch together with the Mediterranean “TYLCVs” clade and an outlier to the sub-Saharan African monopartite begomoviruses infecting tomato (Fig. 1).

**Fig. 2:**
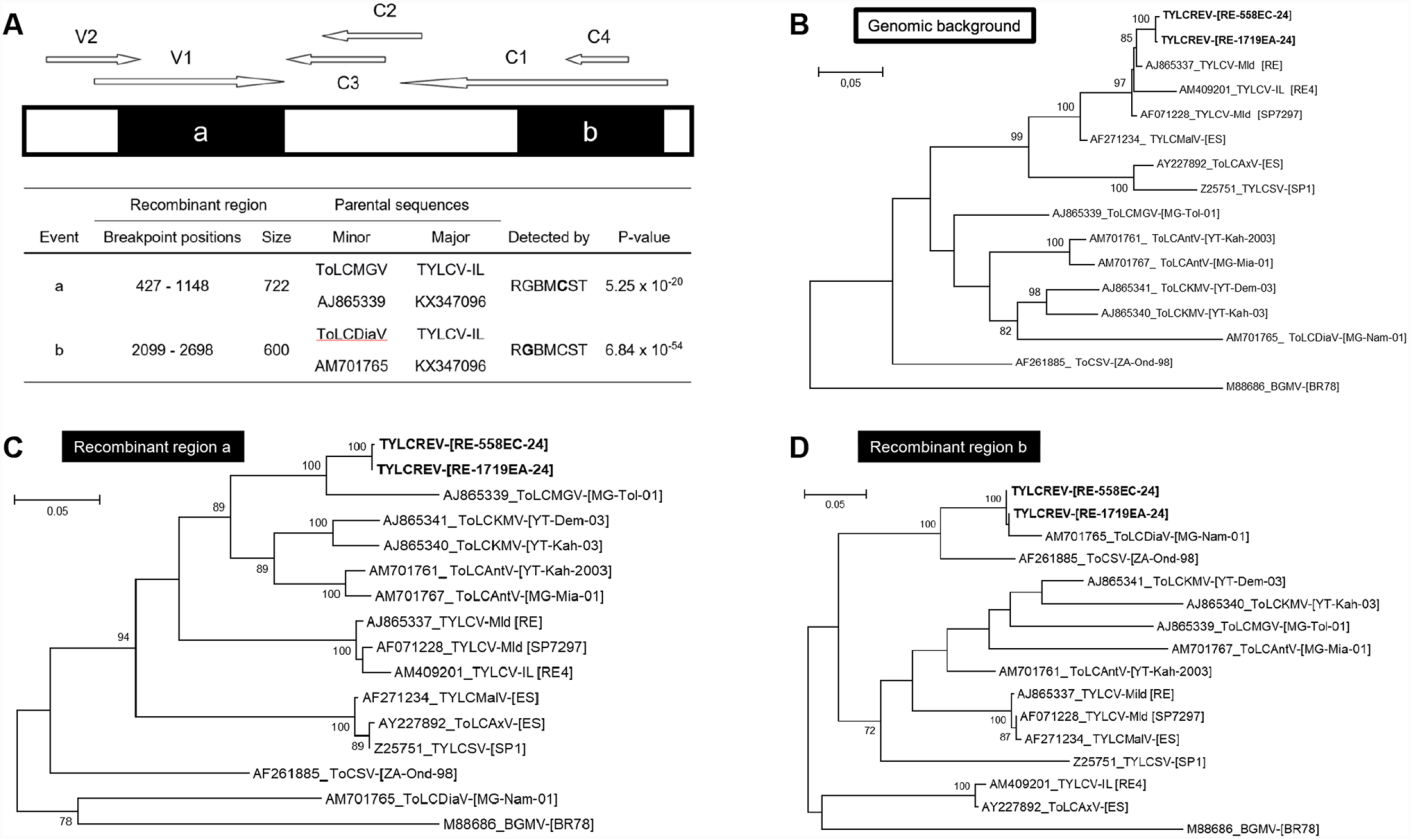
(A) Recombinant regions (a and b) detected within complete DNA-A-like sequences of TYLCREV. Linear schematic representation of the genome organization of TYLCREV. Open reading frames (C1, C2, C3, C4, V1, and V2) encoding proteins > 80 aa are represented by arrows at the top of the diagram. The nucleotide positions of breakpoints are shown in the table. "Parental sequences" are the sequences from the dataset analyzed with the highest similarity to the recombinant sequence. The “minor parent” is the tentative parent of the recombinant region of the sequence. The “major parent” is the apparent contributor of the rest of the sequence. The best *P*-value calculated by the RDP software is provided for each recombination event. (B-D) Maximum-likelihood phylogenetic trees inferred from alignments of (B) the genomic background (positions 1 to 426, 1149 to 2098, and 2699 to 2781 in the TYLCREV genomes), (C) recombinant region a (positions 427 to 1148), and (D) recombinant region b (positions 2099 to 2698). Only bootstrap values higher than 70% are shown (1,000 replicates). For begomovirus abbreviations and accession numbers, see Supplementary Table S2.

Taken together, our results demonstrate that TYLCREV represents a new species in the genus *Begomovirus* and that it resulted from interspecific recombination events between Old World monopartite begomoviruses that are genetically distinct from the current tomato-infecting monopartite begomoviruses described in Africa and the Mediterranean basin. To the best of our knowledge, the three parental lineages of TYLCREV (ToLCMGV, ToLCDiaV, and TYLCV) were reported in distinct geographical areas, raising questions as to where recombination events occurred. The geographical distribution and biological features of this new virus should now be investigated, and the risk of its emergence in tomato fields worldwide should be assessed.

**GenBank accession numbers** The sequences reported in this article have been deposited in the GenBank database under the accession numbers PV126637 (RE-558EC-24) and PV126638 (RE-1719EA-24).

## Supplementary Information

Below is the link to the electronic supplementary material.

Supplementary Fig. S1 Leaf curl symptoms without (A) or with (B) interveinal yellowing associated with tomato yellow leaf curl Reunion virus (genus Begomovirus, family Geminiviridae) observed in February and August 2024 on tomato plants C24-558 (cv. Atitlan) and C24-1719 (cv. Andine Cornue), respectively, in greenhouses in the south of Reunion Island

Supplementary file2 (XLSX 14 KB)

Supplementary file3 (XLS 44 KB)

Supplementary file4 (FAS 5 KB)
