# Supplementary figures and images for: Tomato yellow leaf curl Reunion virus: a novel tomato-infecting monopartite begomovirus from Reunion Island
Source: Arch Virol. 2025 Jul 5;170(8):173. doi: 10.1007/s00705-025-06345-y (PMC12228651; doi:10.1007/s00705-025-06345-y)

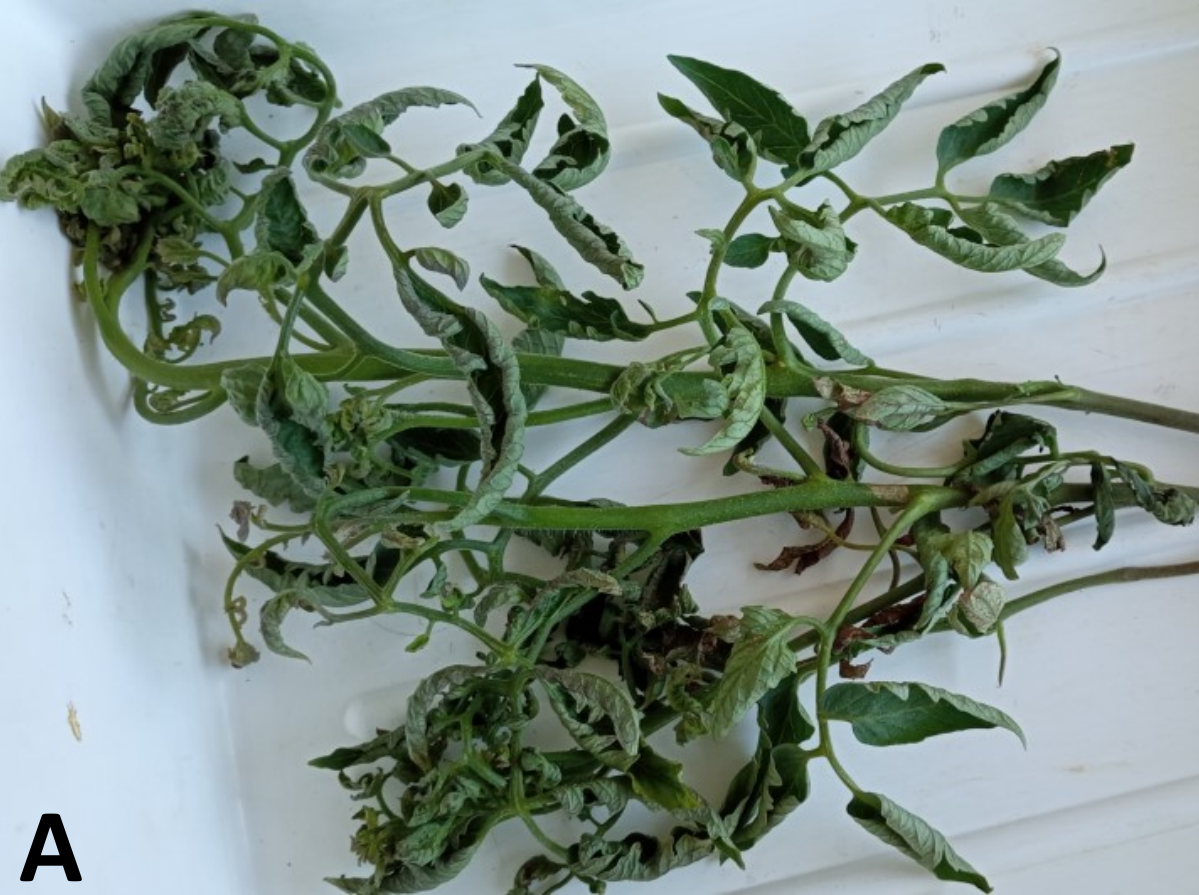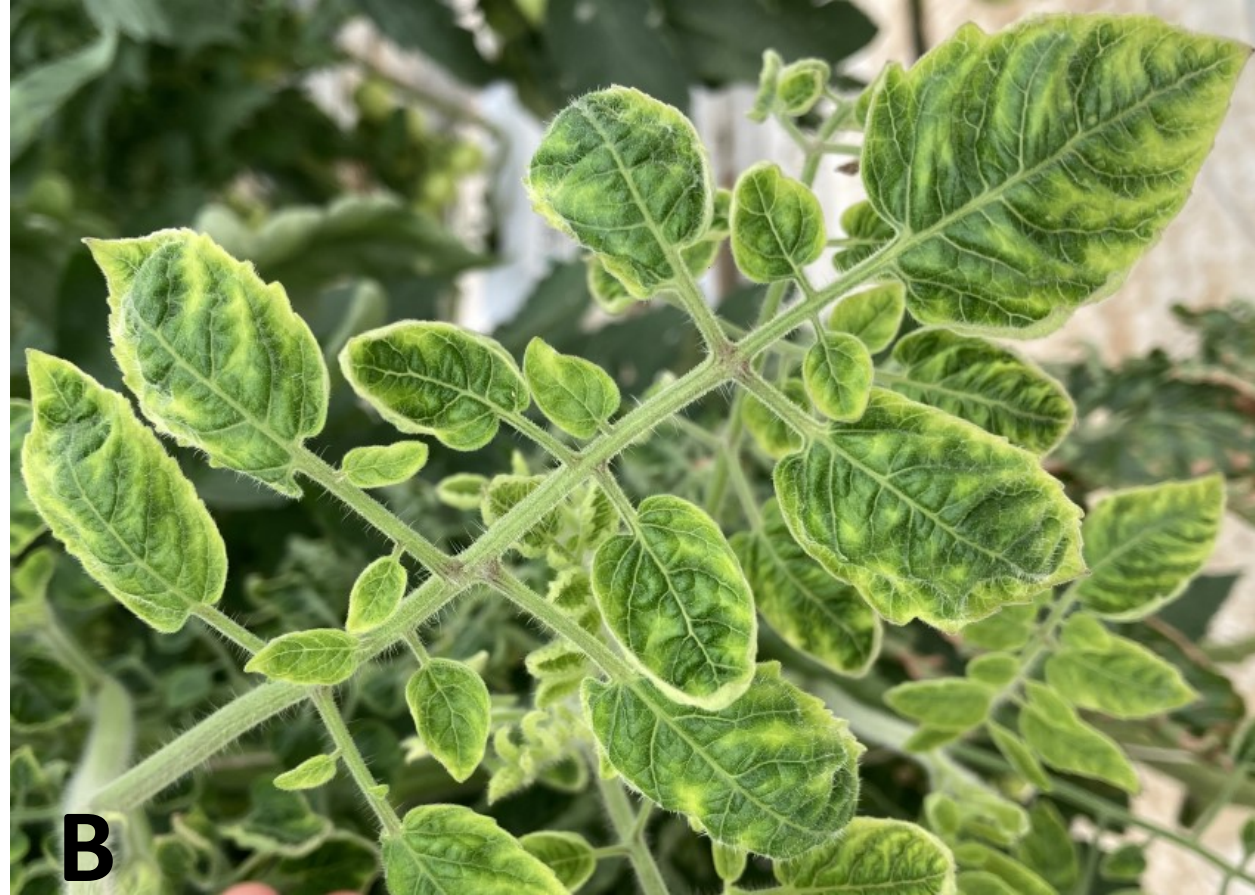

Supplement: Supplementary file 1 — Supplementary Fig. S1 Leaf curl symptoms without (A) or with (B) interveinal yellowing associated with tomato yellow leaf curl Reunion virus (genus Begomovirus, family Geminiviridae) observed in February and August 2024 on tomato plants C24-558 (cv. Atitlan) and C24-1719 (cv. Andine Cornue), respectively, in greenhouses in the south of Reunion Island [file 705_2025_6345_MOESM1_ESM.pdf]
